# Exploring driving behaviors as biomarkers for early identification of individuals at risk for Alzheimer's Disease

**DOI:** 10.1002/alz70857_103413

**Published:** 2025-12-25

**Authors:** Sai Santosh Reddy Danda, Yi Lu Murphy, Amanda Cook Maher, Savannah G Rose, Carol C Persad, Robert A. Koeppe, Bruno Giordani

**Affiliations:** ^1^ University of Michigan‐Dearborn, Dearborn, MI, USA; ^2^ University of Michigan ‐ Dearborn, Dearborn, MI, USA; ^3^ University of Michigan, Ann Arbor, MI, USA; ^4^ University of Michigan Medical School, Ann Arbor, MI, USA

## Abstract

**Background:**

The earliest stages of cognitive decline prodromal to Alzheimer's Disease (AD) are often subtle, becoming more noticeable only during complex tasks, such as driving that demands the integration of cognitive functions, including attention, memory, and decision‐making. Analyzing driving behavior may provide valuable insights into these early cognitive shifts, potentially serving as an indicator of cognitive decline. This study investigates driving patterns in older adults, comparing brain amyloid positive (*Aβ*+) participants—who are at heightened risk of developing AD—to amyloid‐negative (*Aβ*‐) participants to potentially identify behavioral patterns that differentiate these groups.

**Method:**

We evaluated naturalistic driving trips acquired from 30 Aβ+ (aged 68‐81) and 35 Aβ‐ (aged 65‐85) participants. All 65 participants are consensus‐diagnosed cognitively normal older adults. All participants drove their own vehicles for a minimum of 30‐days during which vehicular, physiological signals, and video data were recorded. We analyzed driving attributes including average speed, average acceleration, frequency of large positive and negative jerks, and frequency of deceleration events. Additionally, trip characteristics such as distance, duration, time of day (e.g., overnight, rush hour), and driving maneuvers (left and right turn counts) were included in analyses. Spatial patterns were explored by calculating the number of unique destinations and radius of gyration for each participant. Differences between *Aβ*+ and *Aβ‐* participants were analyzed using independent sample t‐tests.

**Result:**

Significant differences in driving behaviors were evident, such that Aβ+ individuals, who completed more trips on average than Aβ‐ (75.26 vs. 55.34 trips) and demonstrated greater overall driving distance (*p* = 0.049), higher proportion of longer trips exceeding 20 miles (*p* = 0.014), greater radius of gyration (*p* = 0.037), higher counts of large negative jerk events (*p* = 0.023), and fewer deceleration events (*p* = 0.048) compared to Aβ‐ participants. Additionally, Aβ+ participants took fewer left (*p* = 0.022) and right turns (*p* = 0.023) than Aβ‐ participants but visited more unique destinations (*p* = 0.036).

**Conclusion:**

The observed differences in driving patterns between *Aβ+* and *Aβ*− participants may provide valuable insights into early warning signals for subtle cognitive changes related to AD. Future research will focus on developing an intelligent system that leverages significant naturalistic driving features, combined with machine learning techniques, to classify *Aβ+* and *Aβ*− individuals.

